# Are neuropsychiatric symptoms in dementia linked to CSF biomarkers of synaptic and axonal degeneration?

**DOI:** 10.1186/s13195-020-00718-y

**Published:** 2020-11-17

**Authors:** Victor Bloniecki, Henrik Zetterberg, Dag Aarsland, Patrizia Vannini, Hlin Kvartsberg, Bengt Winblad, Kaj Blennow, Yvonne Freund-Levi

**Affiliations:** 1 Department of Neurobiology, Caring Sciences and Society (NVS), Division of Clinical Geriatrics, Karolinska Institutet, Stockholm, Sweden; 2 Department of Dermatology, Karolinska University Hospital, Solna, Sweden; 3 Institute of Neuroscience and Physiology, Department of Psychiatry and Neurochemistry, Sahlgrenska Academy at the University of Gothenburg, Mölndal, Sweden; 4 Clinical Neurochemistry Laboratory, Sahlgrenska University Hospital, Mölndal, Sweden; 5 UK Dementia Research Institute at UCL, London, UK; 6 Department of Neurodegenerative Disease, UCL Institute of Neurology, London, UK; 7 Institute of Psychiatry, Psychology and Neuroscience, King’s College London, London, UK; 8 Center for Age-Related Diseases, Stavanger University Hospital, Stavanger, Norway; 9 Center for Alzheimer Research and Treatment, Department of Neurology, Brigham and Women’s Hospital, Harvard Medical School, Boston, MA USA; 10 Department of Neurobiology, Caring Sciences and Society (NVS), Division of Neurogeriatrics, Karolinska Institutet, Stockholm, Sweden; 11 Theme Aging, Karolinska University Hospital, Huddinge, Sweden; 12 Department of Psychiatry in Region Örebro County and School of Medical Sciences, Faculty of Medicine and Health, Örebro University, Örebro, Sweden; 13 Department of Old Age Psychiatry, Psychology & Neuroscience, King’s College London, London, UK

**Keywords:** NPS, CSF biomarkers, Synaptic and axonal dysfunction, Neurogranin, GAP-43, Neurofilament, Alzheimer’s disease

## Abstract

**Background:**

The underlying disease mechanism of neuropsychiatric symptoms (NPS) in dementia remains unclear. Cerebrospinal fluid (CSF) biomarkers for synaptic and axonal degeneration may provide novel neuropathological information for their occurrence. The aim was to investigate the relationship between NPS and CSF biomarkers for synaptic (neurogranin [Ng], growth-associated protein 43 [GAP-43]) and axonal (neurofilament light [NFL]) injury in patients with dementia.

**Methods:**

A total of 151 patients (mean age ± SD, 73.5 ± 11.0, females *n* = 92 [61%]) were included, of which 64 had Alzheimer’s disease (AD) (34 with high NPS, i.e., Neuropsychiatric Inventory (NPI) score > 10 and 30 with low levels of NPS) and 18 were diagnosed with vascular dementia (VaD), 27 with mixed dementia (MIX), 12 with mild cognitive impairment (MCI), and 30 with subjective cognitive impairment (SCI). NPS were primarily assessed using the NPI. CSF samples were analyzed using enzyme-linked immunosorbent assays (ELISAs) for T-tau, P-tau, Aβ1–42, Ng, NFL, and GAP-43.

**Results:**

No significant differences were seen in the CSF levels of Ng, GAP-43, and NFL between AD patients with high vs low levels of NPS (but almost significantly decreased for Ng in AD patients < 70 years with high NPS, *p* = 0.06). No significant associations between NPS and CSF biomarkers were seen in AD patients. In VaD (*n* = 17), negative correlations were found between GAP-43, Ng, NFL, and NPS.

**Conclusion:**

Our results could suggest that low levels of Ng may be associated with higher severity of NPS early in the AD continuum (age < 70). Furthermore, our data may indicate a potential relationship between the presence of NPS and synaptic as well as axonal degeneration in the setting of VaD pathology.

## Introduction

Neuropsychiatric symptoms (NPS) are common in dementia, greatly impacting the quality of life and caregiver burden [1–3]. The underlying neuropathological mechanisms of NPS are not sufficiently comprehended, but have been linked to region-specific pathology of brain areas, such as the cingulate cortex and left frontal cortex [4, 5]. Additionally, monoaminergic neurotransmission including serotonergic, dopaminergic, or cholinergic dysfunction has been implicated [6–8].

The association between NPS and Alzheimer’s disease (AD) pathology has been investigated in several studies with inconsistent results [9–15]. Cerebrospinal fluid (CSF) biomarkers for synaptic and axonal degeneration have lately been of increased interest in dementia research providing novel information regarding disease neuropathology [16]. However, knowledge regarding the relationship between NPS and synaptic and axonal degeneration is still lacking, and new knowledge in this area could potentially increase our understanding of NPS in dementia disorders. We have previously published data suggesting an association between agitation and tau-associated pathology, as well as axonal degeneration in AD [17].

Synaptic dysfunction is a potential culprit in NPS, and fluorodeoxyglucose positron emission tomography (FDG-PET) imaging studies have indicated that increased NPS levels correlate with synaptic dysfunction in the posterior cingulate cortex, ventromedial prefrontal cortex, and right anterior insula [18]. Synaptic dysfunction in AD is caused by both Aβ and tau pathologies generating an imbalance in synaptic plasticity [19]. Furthermore, synaptic loss is a hallmark of the neurodegenerative process and strongly correlated with both dementia severity and cognitive decline [20–22].

Neurogranin (Ng) is a postsynaptic protein involved in synaptic plasticity and memory formation [23]. Postmortem studies have shown that the full-length Ng is reduced in the parietal and temporal cortex in AD patients and processed into smaller peptides measurable in CSF reflecting synaptic degeneration [24]. CSF Ng levels are elevated in both AD and mild cognitive impairment (MCI) compared to healthy controls and associated with lower cognitive function and deterioration of white matter tracts and predict cognitive decline as well as conversion from MCI to AD [25–36]. No other published study has to our knowledge investigated the association between Ng and NPS.

Growth-associated protein 43 (GAP-43) is a protein primarily distributed in axons and presynaptic terminals involved in neuronal regeneration and formation of neuronal connections [37]. Postmortem studies have shown that GAP-43 is significantly decreased in the frontal cortex of AD patients, while different hippocampal areas are showing both decreased and increased GAP-43 [38–40]. Levels of CSF GAP-43 are increased in AD as compared to both healthy controls and other neurodegenerative disorders, suggesting a specificity for AD [41]. Previous studies have found associations between increased levels of CSF GAP-43 and cognitive decline as well as increased amyloid and tangle burden in the amygdala, cortex, and hippocampus [41–43].

Neurofilament light protein (NFL) is an intermediate filament important for maintaining axonal stability and growth [44, 45]. Axonal damage results in leakage of NFL into CSF making it a marker for ongoing neuroaxonal degeneration [44]. CSF NFL levels are increased in both MCI and AD patients as compared to healthy controls and correlate with both cognitive deterioration and brain atrophy in AD [46–50]. Additionally, CSF NFL is also linked to increased mortality and dementia severity [51].

The primary aim of this study was to investigate the association between NPS and CSF biomarkers for synaptic and axonal degeneration, i.e., levels of CSF Ng, GAP-43, and NFL in AD and other cognitive disorders.

Based on previously published research, we hypothesize that AD patients with a high NPS burden will display increased levels of CSF GAP-43, Ng, and NFL as compared to AD patients with low levels of NPS. AD patients will display higher levels of Ng and GAP-43 as compared to controls, and correlations will be found between Ng, GAP-43, NFL, and NPS within the AD group.

## Methods

### Patients

The study population (*n* = 151) consisted of two separate cohorts, both recruited between 2003 and 2015, from the same center at the memory clinic, Karolinska University Hospital, Stockholm, Sweden.

Cohort 1 consisted of baseline assessments from a previously published randomized controlled trial. Detailed information regarding study population, assessment, and other study specifics can be found in [52, 53]. Briefly, 91 memory-impaired patients with high levels of NPS, defined as a total score of at least 10 points on the Neuropsychiatric Inventory (NPI) [54], and a dementia diagnosis according to the Diagnostic and Statistical Manual of Mental Disorders, Fourth Edition (DSM-IV) [55] or mild cognitive impairment (MCI) were included (37% AD, 30% mixed dementia [MIX], 20% vascular dementia [VaD], 13% MCI). Nine of the 12 patients in the MCI group displayed biomarker abnormalities congruent with underlying AD pathology, i.e., Aβ42/40 ratio levels < 0.063. The study was approved by the Regional Ethics Committee of Karolinska Institutet, Stockholm, Sweden, registration number 441/01.

Diagnostic procedures included somatic, psychiatric, and neurological examinations that were performed by a licensed specialist in geriatric medicine. Neuroimaging (computed tomography) was also performed. In addition to clinical interviews of patients and their caregivers, the standardized scales of cognition (Mini-Mental Status Examination (MMSE)) [56] and neuropsychiatric symptoms (NPI, Cornell Scale for Depression in Dementia) [54, 57] were administered for diagnostic purposes and assessment of behavioral disturbances. Lumbar punctures were successfully performed in 87 out of the 91 included patients at baseline. Lumbar CSF (6 ml) was collected and then stored in polypropylene tubes. The first 2 ml of every sampling was discarded, and the rest was centrifuged at 3000 rpm for 10 min at 4C+ and frozen in aliquots of 2 ml at − 70 °C.

In cohort 2, 60 patients from a total of 13,300 were included from the GEDOC research database and biobank. GEDOC contains data from patients, who had previously been examined and provided informed consent for future research [58]. This included 30 patients diagnosed with subjective cognitive impairment (SCI) and normal CSF AD biomarker profile, defined as Aβ1–42 > 500 ng/ml, T-tau < 400 ng/ml, P-tau < 80 ng/ml. These patients were assessed as cognitively healthy without the presence of any neurodegenerative diseases. Furthermore, 30 AD patients, with a CSF biomarker profile of Aβ1–42 < 500 ng/ml, T-tau > 400 ng/ml, P-tau > 80 ng/ml, and low levels of NPS, were included. Due to the lack of NPI data in this cohort, low NPS was defined by the absence of NPS in the patients’ medical records. Medical records of each patient were reviewed, and subjects were excluded from the study if any significant NPS were mentioned. The clinical assessment at the memory clinic consisted of interviews with patients and caregivers conducted by a specialist in geriatric medicine and research nurses, administration of rating scales for cognition as well as NPS including Cornell Depression Scale, and neuroimaging. CSF samples were collected from all 60 patients. Procedures for lumbar punctures were the same in both cohorts. The GEDOC database is approved by the Regional Ethics Committee of Karolinska Institutet, Stockholm, Sweden, registration number 2011/1987-31/4.

### NPS rating scales

The NPI is a comprehensive rating scale for the assessment of NPS that rates the frequency and severity of 12 major neuropsychiatric and behavioral symptoms in dementia including delusions, hallucinations, dysphoria, anxiety, agitation/aggression, euphoria, disinhibition, irritability/lability, apathy, aberrant motor activity, night-time behavior disturbances, and appetite and eating abnormalities. Each domain can generate a maximum of 12 points, and thus, the total possible score equals 144 points with higher scores indicating a more severe NPS burden. The domains can be assessed individually or as a total NPI score [54]. For the present study, both the total score and the individual subdomains were used.

The Cornell Scale for Depression was used to specifically quantify the depressive symptoms in this study. The scale consists of 19 items assessing various aspects of depressive behaviors with a maximum score of 28 points [57].

#### CSF analyses

CSF Ng concentration was measured using an in-house enzyme-linked immunosorbent assay (ELISA), as described previously in detail [33]. CSF GAP-43 and NFL concentrations were measured by in-house ELISAs, as previously described in detail [41, 59]. CSF levels of T-tau, P-tau, and Aβ1–42 were measured using commercially available INNOTEST ELISAs (Fujirebio Europe, Ghent, Belgium). All CSF samples were handled using the same procedures and analyzed at the Department of Neurochemistry, Mölndal Hospital. All samples were analyzed by board-certified technicians, using one batch of reagents, following strict rules for quality control [60].

#### Statistics

All statistical analyses were done using the Statistica software (version 13). The majority of the included variables in this study (CSF biomarkers) were not normally distributed, and variances between the groups were not equal. Thus, non-parametric statistics were used as the primary analytic method.

Differences in the levels of CSF biomarkers (Ng, GAP-43, NFL) between “AD low NPS” and “AD high NPS” were analyzed using the Mann-Whitney *U* test. Adjusted for age was done by stratification analysis dividing AD patients into three age strata (age < 70, 70–79 and > 80). Spearman rank correlations were used to investigate the associations between biomarkers for synaptic and axonal degeneration (Ng, GAP-43, NFL) and NPS (NPI). Correlation analysis was conducted both on the total cohort and diagnostic subgroups. Data are presented as medians and interquartile range (IQR) if not stated otherwise. A *p* level of < 0.05 was defined as statistically significant. We performed multiple analyses without adjustment in this exploratory study. Any findings therefore need to be interpreted with caution.

## Results

### Baseline data

Baseline clinical characteristics and biomarker levels are provided in Table 1. The mean score for NPI in the AD “high NPS” group was 45.1 points, and the five subdomains with the highest individual NPI ratings included apathy (7.7 points), delusions (6.1 points), anxiety (4.7 points), depression (4.4 points), and appetite/eating disturbances (4.2 points). In cohort 1, NPI was not significantly associated with cognition as measured with MMSE (*r* = − 0.01, *p* = 0.90).

**Table 1:** Descriptive data

	All, ***n*** = 151	AD, ***n*** = 64	AD high NPS, ***n*** = 34, cohort 1	AD low NPS, ***n*** = 30, cohort 2	SCI, ***n*** = 30, cohort 2	MCI, ***N*** = 12, cohort 1	MIX, ***N*** = 27, cohort 1	VaD, ***N*** = 18, cohort 1
**Females (*n*)**	92 (61%)	39 (61%)	20 (59%)	19 (63%)	13 (43%)	10 (83%)	19 (70%)	11 (61%)
**Age, years**	76.0 (18.0)	78.0 (16)^**A**^	79.5 (8.0)^**A,B**^	70.0 (19.0)	60.0 (7.0)	82.0 (6.5)^**A,B**^	83.0 (6.0)^**A,B**^	75.5 (9.0)^**A**^
**MMSE (0–30 points)**	22.0 (7.0)	20.0 (4.5)^**A, C**^	19.5 (6.0)^**A,C**^	21.0 (2.0)^**A**^	30.0 (1.0)	25.5 (2.5)	20.0 (5.0)^**A**^	22.0 (6.0)^**A**^
**CDR (0–3 points)**	1.4 (0.6)	m.d	1.5 (1)	m.d	m.d	1 (0.5)	1.5 (1)	1.4 (1)
**Ng pg/ml**	246 (150)	279 (142)^**A**^	249 (148)	295 (86.0)	175 (85)	314 (223)	244 (177)	218 (119)
**GAP-43 pg/ml**	3643 (2211)	4065 (2077)^**A**^	3901 (2190)	4229 (1818)	2357 (964)	3955 (2586)^**A**^	3639 (2750)^**A**^	3698 (741)^**A**^
**NFL ng/ml**	1530 (1370)	1670 (1360)^**A**^	1970 (1540)	1455 (710)	620 (420)	1770 (1500)^**A**^	2150 (1620)^**A**^	1780 (1800)^**A**^
**Aβ1–42 pg/ml**	460 (280)	410 (33)	440 (170)	396 (84)	805 (200)	490 (250)	435 (160)	460 (220)
**T-tau pg/ml**	630 (430)	698 (391)	680 (400)	719 (470)	265 (92)	730 (430)	695 (220)	550 (330)
**P-tau pg/ml**	85 (56)	98 (37)	87 (52)	104 (31)	45 (18)	99 (48)	97 (39)	74 (33)
**Cornell (0–38 points)**	4.0 (5.0)	4.0 (4.5)	5.0 (5.0)	2.0 (5.0)	4.0 (4.0)	5.5 (5.0)	5.0 (4.0)	6.0 (4.0)
**NPI (0–144 points)**	47 (39)	m.d	42 (29)	m.d	m.d	56 (48)	47 (52)	45.5 (26)

Baseline characteristics of the included patients. Values are presented as medians and interquartile range if not stated otherwise

*m.d* missing data

^**A**^*p* < 0.05 compared to SCI

^**B**^*p* < 0.05 compared to AD low NPS

^**C**^*p* < 0.05 compared to MCI

#### Associations between NPS and markers for synaptic and axonal degeneration

##### Neurogranin

**Fig. 1:**
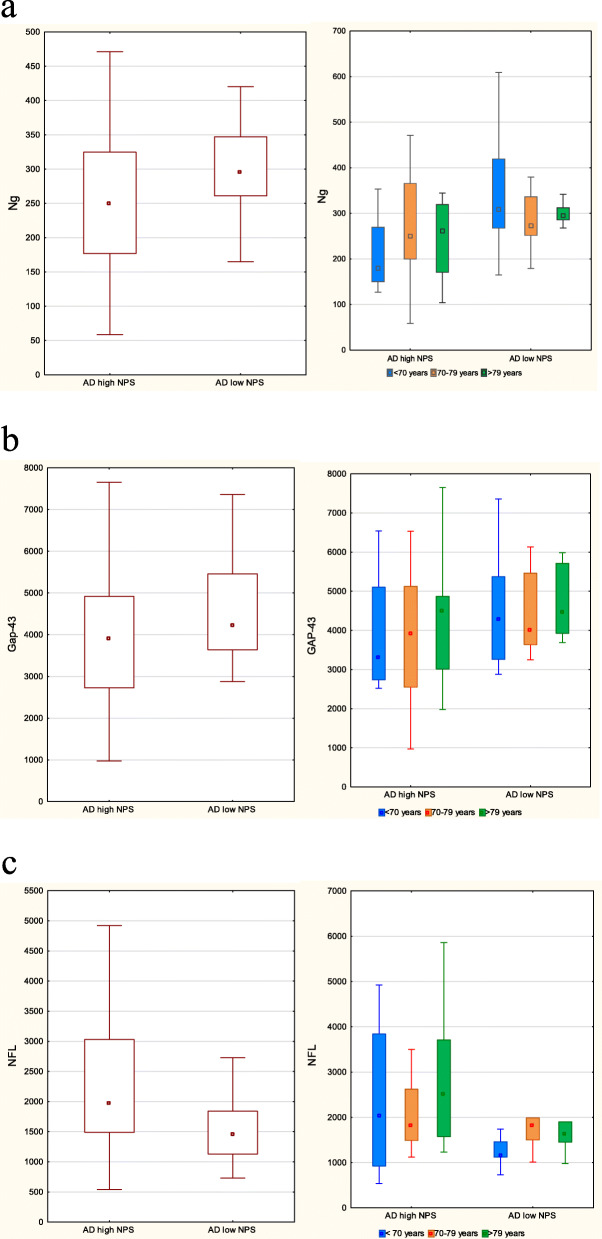
**a** Boxplots comparing the medians of Ng between “AD high NPS” and “AD low NPS.” On the left side including all patients and on the right side divided into age strata. Boxes represent IQ range and whiskers non-outlier maximum and minimum. **b** Boxplots comparing the medians of GAP-43 between “AD high NPS” and “AD low NPS.” On the left side including all patients and on the right side divided into age strata. Boxes represent IQ range and whiskers non-outlier maximum and minimum. **c** Boxplots comparing the medians of NFL between “AD high NPS” and “AD low NPS.” On the left side including all patients and on the right side divided into age strata. Boxes represent IQ range and whiskers non-outlier maximum and minimum

Median CSF Ng levels were significantly higher in AD patients with “low NPS” as compared to “AD high NPS” (*U* = 340, *p* = 0.03, median [Mdn] 295 pg/ml vs 249 pg/ml). After adjustment for age, this difference did not remain significant (but close for patients < 70 years, *p* = 0.06, Mdn 309 pg/ml vs 179 pg/ml) (Fig. 1a).

**Fig. 2:**
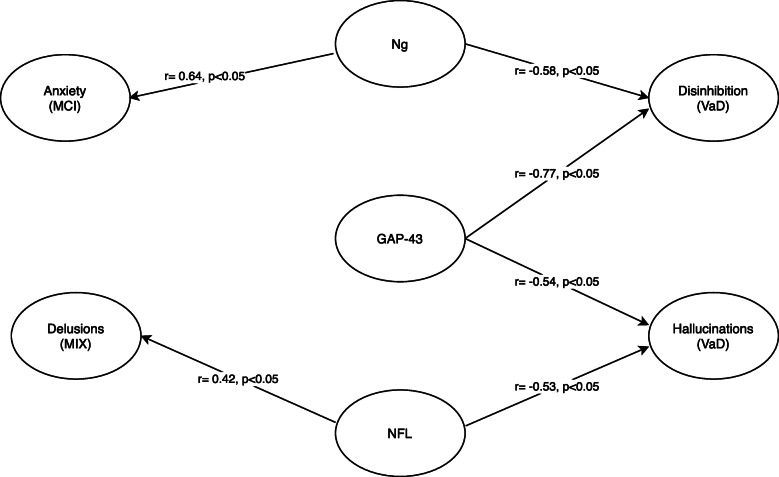
Significant correlations between NPI sub-items and biomarkers for synaptic and axonal injury (*p* < 0.05)

No significant correlations were seen between Ng and NPI total when analyzing all patients, as well as during subgroup analysis depending on dementia diagnosis. When analyzing NPI sub-items, a significant correlation was found between Ng and appetite/eating domain on NPI (*r* = 0.23, *p* = 0.04) in the whole group. Further subgroup analysis depending on diagnosis revealed a negative correlation between disinhibition and Ng in patients with VaD (*r* = − 0.58, *p* = 0.02) as well as a significant positive correlation with anxiety in patients with MCI (*r* = 0.64, *p* = 0.04) (Fig. 2). Ng was not significantly associated with Cornell scores in the whole cohort (*r* = − 0.03, *p* = 0.7) nor in the AD subgroup (*r* = − 0.2, *p* = 0.3).

##### GAP-43

No significant difference in CSF levels of GAP-43 was observed when comparing AD “high NPS” to AD “low NPS” (U = 389.0, *p* = 0.15, Mdn 4229 pg/ml vs 3901 pg/ml) (Fig. 1b).

No significant correlations were found between GAP-43 and NPI total in the whole cohort, as well as during subgroup analysis depending on the diagnosis. When analyzing NPI sub-items, a negative correlation was found between GAP-43 and disinhibition (*r* = − 0.77, *p* = 0.01) as well as hallucinations (*r* = − 0.54, *p* = 0.02) in patients with VaD (Fig. 2). GAP-43 was not significantly associated with Cornell scores in the whole cohort (*r* = − 0.01, *p* = 0.9) nor in the AD subgroup (*r* = − 0.1, *p* = 0.3).

##### Neurofilament

Significantly increased CSF levels of NFL were seen in AD patients with high compared to low levels of NPS (*U* = 284, *p* = 0.01, Mdn 1970 ng/ml vs 1455 ng/ml). Although when adjusting for age, no significant difference between the groups could be observed (Fig. 1c).

No significant correlation was found between CSF NFL levels and NPI-total when analyzing all included patients, as well as during subgroup analysis depending on the diagnosis. Analysis of NPI sub-items revealed a significant negative correlation between NFL and the hallucination domain of NPI (*r* = − 0.53, *p* = 0.03) in VaD patients as well as a significant correlation with the delusion domain of NPI (*r* = 0.42, *p* = 0.03) in MIX patients (Fig. 2). NFL was not significantly associated with Cornell scores in the whole cohort (*r* = 0.04, *p* = 0.6) nor in the AD subgroup (*r* = − 0.1, *p* = 0.5).

## Discussion

The aim of this study was to investigate the relationship between NPS and biomarkers for synaptic and axonal degeneration. No significant differences were observed in CSF levels of Ng, GAP-43, and NFL between AD patients with high vs low levels of NPS, when adjusting for age. However, AD patients younger than 70 years of age with high NPS burden showed a trend towards significantly (*p* = 0.06) decreased levels of Ng as compared to “low NPS” AD patients. Furthermore, no significant correlations were seen between Ng, GAP-43, NFL, and any NPS in AD patients. In contrast, significant negative correlations between NPS of the psychotic spectrum and biomarkers for axonal and synaptic degeneration were observed in patients with VaD.

In contrast with our initial hypothesis, no significant differences in CSF levels of Ng were observed between AD patients with high and low levels of NPS when adjusting for age. A trend towards significantly increased levels of Ng was observed in younger patients (< 70 years of age) with AD and low levels of NPS. Ng has previously been suggested to increase as an adaptive response to neurodegeneration and synaptic loss in the early stages of AD [43]. Thus, this finding could speculatively indicate that higher levels of Ng reflect an adequate neuroprotective response associated with less severe NPS in the context of early AD. We could not observe any significant differences in GAP-43 levels between AD patients with high and low NPS supporting that synaptic dysfunction, as measured by CSF levels of by GAP-43 and Ng, is not clearly associated with the presence of NPS.

Similarly, after adjustment for age, CSF levels of NFL did not differ between AD patients with high and low levels of NPS, indicating that the overall level of axonal degeneration might not be associated with the presence of NPS. This finding is also contrary to our initial hypothesis, and to our knowledge, the relationship between NFL and NPS has not been examined in earlier studies. Conceptually, an increase was expected due to previous research indicating NFL as a marker for subcortical axonal neurodegeneration associated with both cognitive deterioration and longitudinal brain atrophy affecting the areas involved in controlling behavior, such as the hippocampus [47, 48]. Possibly, this finding could be explained by the fact that region-specific axonal degeneration may be of importance for the development of NPS rather than the overall level of axonal degeneration as measured with CSF NFL.

When analyzing all AD patients, no significant correlations between any of the biomarkers Ng, GAP-43, NFL, and NPS were observed. Several explanations for these findings are possible, e.g., that synaptic and axonal degeneration is not involved in the neuropathology of NPS. However, given the pivotal role of synaptic dysfunction in dementia, this seems unlikely [61]. Previous studies have shown that Ng remains stable during longitudinal follow-up in patients with AD [25]. This might suggest that the increase in Ng occurs early in the disease progression, and associations may be difficult to identify, depending on when a measurement occurs in the disease continuum. We found a strong association between Ng and anxiety in patients with MCI, potentially indicating that increased synaptic dysfunction might be associated with NPS of anxiety type. Although the relationship between CSF Ng and NPS has not been previously investigated, this finding is conceptually in line with earlier studies showing that Ng is elevated in MCI and predicts cognitive deterioration [25–27]. Of interest, an animal study using Ng knockout mice showed behavioral changes including increased anxiety and decreased stress tolerance supporting the role of Ng in the pathophysiology of NPS [62].

In patients with VaD, we found several strong negative correlations between synaptic and axonal biomarkers and psychotic behavior as well as disinhibition. CSF Ng levels were associated with disinhibition, and GAP-43 levels displayed negative correlations with both hallucinations and disinhibition, while NFL was associated with hallucinations. This may imply that in the setting of vascular pathology, synaptic and axonal dysfunction is associated with NPS of the psychotic spectrum as well as disinhibition. Of interest, synaptic loss and dysfunction are a core feature in the development of psychotic disorders such as schizophrenia in non-demented patients [63, 64]. Mutations in the gene encoding Ng as well as alterations of GAP-43 levels in the hippocampus have also been associated with schizophrenia [65, 66]. Furthermore, it has recently been demonstrated that NFL has an important regulatory role in the function of dendritic spines through interaction with subunits of the NMDA receptor and that reduction of neuronal NFL generates psychotic behavioral disturbances via reduced NMDA receptor activity [67]. Overall, there are several indications that pathological alterations of these proteins are involved in the pathogenesis of psychotic disturbances.

Some conceptual problems arise when considering that our observed correlations in patients with VaD are all negative. The classical viewpoint is that increased CSF levels of Ng, NFL, and GAP-43 represent a pathological state, whereas our results infer the reverse relationship, i.e., high levels of these CSF biomarkers are associated with low levels of NPS symptoms. Since CSF biomarker measurements reflect the total output from ongoing neurobiological processes, and for example, GAP-43 has been shown to display both region-specific increases and decreases in the context of neurodegeneration [39, 40, 43], interpretation of this becomes highly difficult. Additionally, studies have shown that CSF GAP-43 concentration increase transiently over a period of approximately 5 months during cerebral ischemia [68] and then returns to baseline values, making the interpretation of these findings in VaD even more difficult. However, one could hypothesize that since Ng in patients with VaD is decreased as compared to healthy controls [69], low levels of Ng and GAP-43 in CSF should be considered reflective of an increase in neuropathology in the setting of VaD. Conversely, high levels could be a consequence of an adequate adaptive response to synaptic degeneration resulting in less NPS.

Interestingly, one previous study has shown that high levels of Ng among Aβ− subjects are protective against cognitive decline in the setting of MCI, while it is associated with increased cognitive deterioration in AD patients [70]. This further supports the notion that increased CSF Ng may be associated with different biological mechanisms during dementia progression.

### Limitations

Several limitations exist in this study. Our sample size during subgroup analysis is relatively small, thus increasing the risk of type 2 errors. We conducted multiple correlation analyses in this study, thus increasing the risk of type 1 errors. All results must therefore be interpreted with caution, and replication is needed. We have also combined two different cohorts increasing the heterogeneity of the population. We have also divided the AD group into “high” and “low” NPS without any quantitative measure for the “low NPS” group. Patients in the “high NPS” group were specifically selected based on the presence of dementia and high levels of NPS, defined as NPI > 10, whereas the “low NPS” group was sampled from the GEDOC database at Karolinska University Hospital, and unfortunately, no values for the NPI could be obtained. Instead, the individual medical records of participants were obtained and thoroughly searched for indications of NPS by reading journal entries written by a specialist in geriatric medicine, neuropsychologist, occupational therapists, and curators as well assessments of other rating scales. Patients were subsequently excluded if there were any signs of significant amounts of NPS. Additionally, the Cornell score was used as a proxy due to the overlap in several testing items between Cornell and NPI providing further support of a significant difference with regard to NPS between the groups.

## Conclusions

In summary, we have found some evidence potentially implicating synaptic and axonal dysfunction in the promotion of NPS. Further research is needed to clearly determine these relationships and evaluate the potential utility of these biomarkers in a clinical setting.

## Data Availability

The datasets analyzed during the current study are available from the corresponding author on reasonable request.

## Abbreviations

AD: Alzheimer’s disease
CSF: Cerebrospinal fluid
ELISAs: Enzyme-linked immunosorbent assays
GAP-43: Growth-associated protein 43
Mdn: Median
M: Mean
MIX: Mixed dementia
MCI: Mild cognitive impairment
MMSE: Mini-Mental State Examination
NFL: Neurofilament light
Ng: Neurogranin
NPS: Neuropsychiatric symptoms
NPI: Neuropsychiatric inventory
SCI: Subjective cognitive impairment
VaD: Vascular dementia

## References

[1] Lyketsos CG, Carrillo MC, Ryan JM, Khachaturian AS, Trzepacz P, Amatniek J (2011). Neuropsychiatric symptoms in Alzheimer’s disease. Alzheimers Dement.

[2] Vik-Mo AO, Giil LM, Ballard C, Aarsland D (2018). Course of neuropsychiatric symptoms in dementia: 5-year longitudinal study. Int J Geriatr Psychiatry.

[3] Kochhann R, Borba E, Cerveira MO, Onyszko D, de Jesus A, Forster L (2011). Neuropsychiatric symptoms as the main determinant of caregiver burden in Alzheimer’s disease. Dement Neuropsychol.

[4] Hu X, Meiberth D, Newport B, Jessen F (2015). Anatomical correlates of the neuropsychiatric symptoms in Alzheimer’s disease. Curr Alzheimer Res.

[5] Bruen PD, McGeown WJ, Shanks MF, Venneri A (2008). Neuroanatomical correlates of neuropsychiatric symptoms in Alzheimer’s disease. Brain.

[6] Niederkofler V, Asher TE, Okaty BW, Rood BD, Narayan A, Hwa LS (2016). Identification of serotonergic neuronal modules that affect aggressive behavior. Cell Rep.

[7] Lanari A, Amenta F, Silvestrelli G, Tomassoni D, Parnetti L (2006). Neurotransmitter deficits in behavioural and psychological symptoms of Alzheimer’s disease. Mech Ageing Dev.

[8] Choudhury A, Sahu T, Ramanujam PL, Banerjee AK, Chakraborty I, Kumar RA (2018). Neurochemicals, behaviours and psychiatric perspectives of neurological diseases. Neuropsychiatry (London).

[9] Engelborghs S, Maertens K, Vloeberghs E, Aerts T, Somers N, Mariën P (2006). Neuropsychological and behavioural correlates of CSF biomarkers in dementia. Neurochem Int.

[10] Ramakers IHGBGB, Verhey FRJJ, Scheltens P, Hampel H, Soininen H, Aalten P (2013). Anxiety is related to Alzheimer cerebrospinal fluid markers in subjects with mild cognitive impairment. Psychol Med.

[11] Kuo HC, Yen HC, Huang CC, Hsu WC, Wei HJ, Lin CL (2015). Cerebrospinal fluid biomarkers for neuropsychological symptoms in early stage of late-onset Alzheimer’s disease. Int J Neurosci.

[12] Roe CM, Fagan AM, Grant EA, Holtzman DM, Morris JC (2013). CSF biomarkers of Alzheimer disease. Neurology..

[13] Skogseth R, Mulugeta E, Ballard C, Rongve A, Nore S, Alves G (2008). Neuropsychiatric correlates of cerebrospinal fluid biomarkers in Alzheimer’s disease. Dement Geriatr Cogn Disord.

[14] Kramberger MG, Jelic V, Kåreholt I, Enache D, Eriksdotter Jönhagen M, Winblad B (2012). Cerebrospinal fluid Alzheimer markers in depressed elderly subjects with and without Alzheimer’s disease. Dement Geriatr Cogn Dis Extra.

[15] Koppel J, Sunday S, Buthorn J, Goldberg T, Davies P, Greenwald B (2013). Elevated CSF tau is associated with psychosis in Alzheimer’s disease. Am J Psychiatry.

[16] Blennow K, Zetterberg H (2018). Biomarkers for Alzheimer’s disease: current status and prospects for the future. J Intern Med.

[17] Bloniecki V, Aarsland D, Cummings J, Blennow K, Freund-Levi Y (2014). Agitation in dementia: relation to core cerebrospinal fluid biomarker levels. Dement Geriatr Cogn Dis Extra.

[18] Ng KP, Pascoal TA, Mathotaarachchi S, Chung C-O, Benedet AL, Shin M (2017). Neuropsychiatric symptoms predict hypometabolism in preclinical Alzheimer disease. Neurology..

[19] Marttinen M, Kurkinen KM, Soininen H, Haapasalo A, Hiltunen M. Synaptic dysfunction and septin protein family members in neurodegenerative diseases. Mol Neurodegener. 2015;10:16. 10.1186/s13024-015-0013-z PMC4391194 25888325

[20] Clare R, King VG, Wirenfeldt M, Vinters HV (2010). Synapse loss in dementias. J Neurosci Res.

[21] Shankar GM, Walsh DM (2009). Alzheimer’s disease: synaptic dysfunction and Aβ. Mol Neurodegener.

[22] Skaper SD, Facci L, Zusso M, Giusti P (2017). Synaptic plasticity, dementia and Alzheimer disease. CNS Neurol Disord Drug Targets.

[23] Jones KJ, Templet S, Zemoura K, Kuzniewska B, Pena FX, Hwang H (2018). Rapid, experience-dependent translation of neurogranin enables memory encoding. Proc Natl Acad Sci.

[24] Kvartsberg H, Lashley T, Murray CE, Brinkmalm G, Cullen NC, Höglund K (2019). The intact postsynaptic protein neurogranin is reduced in brain tissue from patients with familial and sporadic Alzheimer’s disease. Acta Neuropathol.

[25] Kester MI, Teunissen CE, Crimmins DL, Herries EM, Ladenson JKH, Scheltens P (2015). Neurogranin as a cerebrospinal fluid biomarker for synaptic loss in symptomatic Alzheimer disease. JAMA Neurol.

[26] Hellwig K, Kvartsberg H, Portelius E, Andreasson U, Oberstein TJ, Lewczuk P (2015). Neurogranin and YKL-40: independent markers of synaptic degeneration and neuroinflammation in Alzheimer’s disease. Alzheimers Res Ther.

[27] Wang L (2019). Association of cerebrospinal fluid neurogranin with Alzheimer’s disease. Aging Clin Exp Res.

[28] Kvartsberg H, Duits FH, Ingelsson M, Andreasen N, Öhrfelt A, Andersson K (2015). Cerebrospinal fluid levels of the synaptic protein neurogranin correlates with cognitive decline in prodromal Alzheimer’s disease. Alzheimers Dement.

[29] Portelius E, Zetterberg H, Skillbäck T, Törnqvist U, Andreasson U, Trojanowski JQ (2015). Cerebrospinal fluid neurogranin: relation to cognition and neurodegeneration in Alzheimer’s disease. Brain..

[30] Headley A, De Leon-Benedetti A, Dong C, Levin B, Loewenstein D, Camargo C (2018). Neurogranin as a predictor of memory and executive function decline in MCI patients. Neurology..

[31] Kim WH, Racine AM, Adluru N, Hwang SJ, Blennow K, Zetterberg H (2019). Cerebrospinal fluid biomarkers of neurofibrillary tangles and synaptic dysfunction are associated with longitudinal decline in white matter connectivity: a multi-resolution graph analysis. NeuroImage Clin.

[32] Wellington H, Paterson RW, Portelius E, Törnqvist U, Magdalinou N, Fox NC (2016). Increased CSF neurogranin concentration is specific to Alzheimer disease. Neurology..

[33] Portelius E, Olsson B, Höglund K, Cullen NC, Kvartsberg H, Andreasson U (2018). Cerebrospinal fluid neurogranin concentration in neurodegeneration: relation to clinical phenotypes and neuropathology. Acta Neuropathol.

[34] Mattsson N, Insel PS, Palmqvist S, Portelius E, Zetterberg H, Weiner M (2016). Cerebrospinal fluid tau, neurogranin, and neurofilament light in Alzheimer’s disease. EMBO Mol Med.

[35] Dhiman K, Blennow K, Zetterberg H, Martins RN, Gupta VB (2019). Cerebrospinal fluid biomarkers for understanding multiple aspects of Alzheimer’s disease pathogenesis. Cell Mol Life Sci.

[36] Sanfilippo C, Forlenza O, Zetterberg H, Blennow K (2016). Increased neurogranin concentrations in cerebrospinal fluid of Alzheimer’s disease and in mild cognitive impairment due to AD. J Neural Transm.

[37] Sjögren M, Minthon L, Davidsson P, Granérus A-KK-K, Clarberg A, Vanderstichele H (2000). CSF levels of tau, β-amyloid 1-42 and GAP-43 in frontotemporal dementia, other types of dementia and normal aging. J Neural Transm.

[38] Davidsson P, Blennow K (1998). Neurochemical dissection of synaptic pathology in Alzheimer’s disease. Int Psychogeriatrics.

[39] Bogdanovic N, Davidsson P, Volkmann I, Winblad B, Blennow K (2000). Growth-associated protein GAP-43 in the frontal cortex and in the hippocampus in Alzheimer’s disease: an immunohistochemical and quantitative study. J Neural Transm.

[40] Rekart JL, Quinn B, Mesulam MM, Routtenberg A (2004). Subfield-specific increase in brain growth protein in postmortem hippocampus of Alzheimer’s patients. Neuroscience..

[41] Sandelius Å, Portelius E, Källén Å, Zetterberg H, Rot U, Olsson B (2019). Elevated CSF GAP-43 is Alzheimer’s disease specific and associated with tau and amyloid pathology. Alzheimers Dement.

[42] Sjögren M, Davidsson P, Gottfries J, Vanderstichele H, Edman Å, Vanmechelen E (2001). The cerebrospinal fluid levels of tau, growth-associated protein-43 and soluble amyloid precursor protein correlate in Alzheimer’s disease, reflecting a common pathophysiological process. Dement Geriatr Cogn Disord.

[43] Remnestål J, Just D, Mitsios N, Fredolini C, Mulder J, Schwenk JM (2016). CSF profiling of the human brain enriched proteome reveals associations of neuromodulin and neurogranin to Alzheimer’s disease. Proteomics Clin Appl.

[44] Khalil M, Teunissen CE, Otto M, Piehl F, Sormani MP, Gattringer T (2018). Neurofilaments as biomarkers in neurological disorders. Nat Rev Neurol.

[45] Gaetani L, Blennow K, Calabresi P, Di Filippo M, Parnetti L, Zetterberg H (2019). Neurofilament light chain as a biomarker in neurological disorders. J Neurol Neurosurg Psychiatry.

[46] Lin Y-S, Lee W-J, Wang S-J, Fuh J-L (2018). Levels of plasma neurofilament light chain and cognitive function in patients with Alzheimer or Parkinson disease. Sci Rep.

[47] Zetterberg H, Skillbäck T, Mattsson N, Trojanowski JQ, Portelius E, Shaw LM (2016). Association of cerebrospinal fluid neurofilament light concentration with Alzheimer disease progression. JAMA Neurol.

[48] Olsson B, Portelius E, Cullen NC, Sandelius Å, Zetterberg H, Andreasson U (2019). Association of cerebrospinal fluid neurofilament light protein levels with cognition in patients with dementia, motor neuron disease, and movement disorders. JAMA Neurol.

[49] Merluzzi AP, Vogt NM, Norton D, Jonaitis E, Clark LR, Carlsson CM (2019). Differential effects of neurodegeneration biomarkers on subclinical cognitive decline. Alzheimers Dement Transl Res Clin Interv.

[50] Lewczuk P, Ermann N, Andreasson U, Schultheis C, Podhorna J, Spitzer P (2018). Plasma neurofilament light as a potential biomarker of neurodegeneration in Alzheimer’s disease. Alzheimers Res Ther.

[51] Skillbäck T, Farahmand B, Bartlett JW, Rosén C, Mattsson N, Nägga K (2014). CSF neurofilament light differs in neurodegenerative diseases and predicts severity and survival. Neurology.

[52] Freund-Levi Y, Jedenius E, Tysen-Bäckström AC, Lärksäter M, Wahlund LO, Eriksdotter M (2014). Galantamine versus risperidone treatment of neuropsychiatric symptoms in patients with probable dementia: an open randomized trial. Am J Geriatr Psychiatry.

[53] Freund-Levi Y, Bloniecki V, Auestad B, Bäckström ACT, Lärksäter M, Aarsland D (2014). Galantamine versus risperidone for agitation in people with dementia: a randomized, twelve-week, single-center study. Dement Geriatr Cogn Disord.

[54] Cummings JL, Mega M, Gray K, Rosenberg-Thompson S, Carusi DA, Gornbein J (1994). The neuropsychiatric inventory: comprehensive assessment of psychopathology in dementia. Neurology..

[55] First MB, Frances A, Pincus HA (2002). DSM-IV-TR handbook of differential diagnosis [internet]. DSM-IV-TR handbook of differential diagnosis.

[56] Folstein MF, Folstein SE, McHugh PR (1975). “Mini-mental state”. A practical method for grading the cognitive state of patients for the clinician. J Psychiatr Res.

[57] Alexopoulos GS, Abrams RC, Young RC, Shamoian CA (1988). Cornell Scale for Depression in dementia. Biol Psychiatry.

[58] Miley-Akerstedt A, Jelic V, Marklund K, Walles H, Åkerstedt T, Hagman G (2018). Lifestyle factors are important contributors to subjective memory complaints among patients without objective memory impairment or positive neurochemical biomarkers for Alzheimer’s disease. Orig Res Artic Dement Geriatr Cogn Disord Extra.

[59] Gaetani L, Höglund K, Parnetti L, Pujol-Calderon F, Becker B, Eusebi P (2018). A new enzyme-linked immunosorbent assay for neurofilament light in cerebrospinal fluid: analytical validation and clinical evaluation. Alzheimers Res Ther.

[60] Palmqvist S, Zetterberg H, Blennow K, Vestberg S, Andreasson U, Brooks DJ (2014). Accuracy of brain amyloid detection in clinical practice using cerebrospinal fluid β-amyloid 42: a cross-validation study against amyloid positron emission tomography. JAMA Neurol.

[61] Chen Y, Fu AKY, Ip NY (2019). Synaptic dysfunction in Alzheimer’s disease: mechanisms and therapeutic strategies. Pharmacol Ther.

[62] Miyakawa T, Yared E, Pak JH, Huang FL, Huang K-P, Crawley JN (2001). Neurogranin null mutant mice display performance deficits on spatial learning tasks with anxiety related components. Hippocampus..

[63] Yin DM, Chen YJ, Sathyamurthy A, Xiong WC, Mei L (2012). Synaptic dysfunction in schizophrenia. Adv Exp Med Biol.

[64] Osimo EF, Beck K, Reis Marques T, Howes OD (2019). Synaptic loss in schizophrenia: a meta-analysis and systematic review of synaptic protein and mRNA measures. Mol Psychiatry.

[65] Ruano D, Aulchenko YS, Macedo A, Soares MJ, Valente J, Azevedo MH (2008). Association of the gene encoding neurogranin with schizophrenia in males. J Psychiatr Res.

[66] Chambers JS, Thomas D, Saland L, Neve RL, Perrone-Bizzozero NI (2005). Growth-associated protein 43 (GAP-43) and synaptophysin alterations in the dentate gyrus of patients with schizophrenia. Prog Neuropsychopharmacol Biol Psychiatry.

[67] Yuan A, Veeranna, Sershen H, Basavarajappa BS, Smiley JF, Hashim A (2018). Neurofilament light interaction with GluN1 modulates neurotransmission and schizophrenia-associated behaviors. Transl Psychiatry.

[68] Sandelius Å, Cullen NC, Källén Å, Rosengren L, Jensen C, Kostanjevecki V (2018). Transient increase in CSF GAP-43 concentration after ischemic stroke. BMC Neurol.

[69] Janelidze S, Hertze J, Zetterberg H, Landqvist Waldö M, Santillo A, Blennow K (2016). Cerebrospinal fluid neurogranin and YKL-40 as biomarkers of Alzheimer’s disease. Ann Clin Transl Neurol.

[70] Bos I, Vos S, Verhey F, Scheltens P, Teunissen C, Engelborghs S (2019). Cerebrospinal fluid biomarkers of neurodegeneration, synaptic integrity, and astroglial activation across the clinical Alzheimer’s disease spectrum. Alzheimers Dement.

